# Bis(μ-6-hydroxy­naphthalene-1-carboxyl­ato)bis­[(6-hydroxy­naphthalene-1-car­box­yl­ato)(1,10-phenanthroline)cadmium(II)] tetra­hydrate

**DOI:** 10.1107/S1600536809043475

**Published:** 2009-10-28

**Authors:** Chun-Sen Liu, Min Hu, Liang-Qi Guo

**Affiliations:** a Zhengzhou University of Light Industry, Henan Provincial Key Laboratory of Surface & Interface Science, Henan, Zhengzhou 450002, People’s Republic of China

## Abstract

The title complex, [Cd_2_(C_11_H_7_O_3_)_4_(C_12_H_8_N_2_)_2_]·4H_2_O, has a centrosymmetric binuclear structure in which two Cd^II^ atoms are both six-coordinated and bridged by 6-hydroxy­naphthalene-1-carboxyl­ate ligands, with a Cd⋯Cd separation of 3.671 (1) Å. The remaining coordination sites are occupied by two N atoms of a 1,10-phenanthroline ligand and two O atoms of a 6-hydroxy­naphthalene-1-carboxyl­ate ligand. The crystal packing is stabilized by O—H⋯O hydrogen-bonding inter­actions.

## Related literature

For the preparation of functional coordination architectures, see: Barnett & Champness (2003 ▶); Comba & Schiek (2003 ▶); Telfer & Kuroda (2003 ▶); Robin & Fromm (2006 ▶); Tranchemontagne *et al.* (2009 ▶). For complexes with carboxylic acid ligands, see: Bania *et al.* (2007 ▶); Liu *et al.* (2006 ▶); Marsh (2004 ▶); Paz & Klinowski (2004 ▶); Qin *et al.* (2008 ▶); Shi *et al.* (2005 ▶); Wu *et al.* (2006 ▶); Xu *et al.* (2005 ▶); Ye *et al.* (2005 ▶).
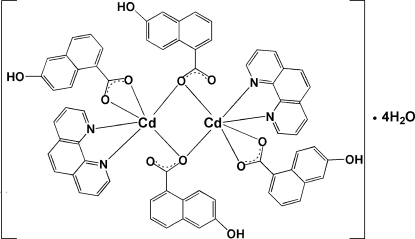


## Experimental

### 

#### Crystal data

[Cd_2_(C_11_H_7_O_3_)_4_(C_12_H_8_N_2_)_2_]·4H_2_O
*M* *_r_* = 1405.96
Monoclinic, 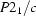

*a* = 11.7382 (11) Å
*b* = 15.1433 (14) Å
*c* = 18.2059 (13) Å
β = 116.430 (4)°
*V* = 2897.9 (4) Å^3^
*Z* = 2
Mo *K*α radiation
μ = 0.81 mm^−1^
*T* = 296 K
0.28 × 0.21 × 0.18 mm

#### Data collection

Bruker SMART CCD area-detector diffractometer
Absorption correction: multi-scan (*SADABS*; Sheldrick, 1996 ▶) *T*_min_ = 0.804, *T*_max_ = 0.868
20423 measured reflections
5096 independent reflections
4064 reflections with *I* > 2σ(*I*)
*R*_int_ = 0.037

#### Refinement

*R*[*F* ^2^ > 2σ(*F* ^2^)] = 0.028
*wR*(*F* ^2^) = 0.063
*S* = 1.03
5096 reflections
406 parameters
H-atom parameters constrained
Δρ_max_ = 0.30 e Å^−3^
Δρ_min_ = −0.41 e Å^−3^

### 

Data collection: *SMART* (Bruker, 2007 ▶); cell refinement: *SAINT* (Bruker, 2007 ▶); data reduction: *SAINT*; program(s) used to solve structure: *SHELXS97* (Sheldrick, 2008 ▶); program(s) used to refine structure: *SHELXL97* (Sheldrick, 2008 ▶); molecular graphics: *SHELXTL* (Sheldrick, 2008 ▶); software used to prepare material for publication: *SHELXTL* and *PLATON* (Spek, 2009 ▶).

## Supplementary Material

Crystal structure: contains datablocks I, global. DOI: 10.1107/S1600536809043475/bt5107sup1.cif

Structure factors: contains datablocks I. DOI: 10.1107/S1600536809043475/bt5107Isup2.hkl

Additional supplementary materials:  crystallographic information; 3D view; checkCIF report

## Figures and Tables

**Table 1:** Hydrogen-bond geometry (Å, °)

*D*—H⋯*A*	*D*—H	H⋯*A*	*D*⋯*A*	*D*—H⋯*A*
O1*W*—H1⋯O5^i^	0.85	2.15	2.934 (3)	154
O1*W*—H2⋯O1^ii^	0.85	2.03	2.872 (3)	170
O2*W*—H3⋯O1*W*	0.85	1.96	2.801 (4)	170
O2*W*—H4⋯O2	0.85	2.08	2.883 (3)	156
O5—H5*B*⋯O4^iii^	0.82	1.84	2.664 (3)	176
O6—H6*B*⋯O2*W*^iv^	0.82	1.91	2.728 (4)	180

Symmetry codes: (i) 
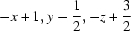
; (ii) 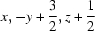; (iii) 
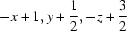
; (iv) 
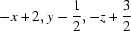
.

**Fig. 1.**
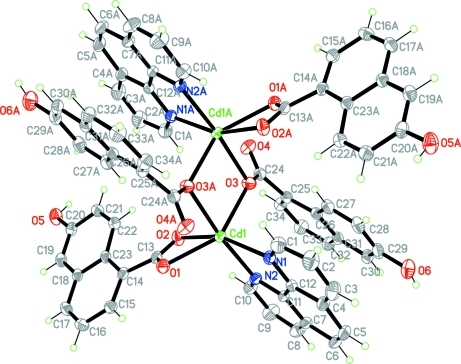
The molecular structure of the title complex. Displacement ellipsoids are drawn at the 30% probability level. Atoms labelled with the suffix A are generated by the symmetry operation (1 – x, 1 – y, 1 – z). Four free water molecules are omitted for clarity.

## supplementary crystallographic information

### Comment

Metallosupramolecular species built from transition metal ions and organic bridging ligands have been rapidly developed in recent years because of their fascinating structural diversities and potential applications as functional materials (Barnett & Champness, 2003; Comba & Schiek, 2003; Telfer & Kuroda, 2003; Robin & Fromm, 2006; Tranchemontagne *et al.*, 2009). The effective and facile approach for the synthesis of such complexes is still the appropriate choice of well designed organic ligands as bridges or terminal groups with metal ions as nodes. Among various ligands, the versatile carboxylic acid ligands, especially for the benzene- and naphthalene-based di- and multi-carboxylic acids, have been most widely employed in the preparation of various Cd^II^–carboxylate complexes (Marsh, 2004; Paz & Klinowski, 2004; Qin *et al.*, 2008; Shi *et al.*, 2005; Wu *et al.*, 2006; Xu *et al.*, 2005). In contrast, the skillful use of monocarboxylic acid ligands with the naphthalene skeleton to construct functional Cd^II^–carboxylate compounds has been less investigated to date (Bania *et al.*, 2007; Liu *et al.*, 2006). In addition, the introduction of 2,2'-bipyridyl-like bidentate chelating molecules, such as 1,10-phenanthroline or 2,2'-bipyridine, as an auxiliary co-ligand into the reaction systems involving carboxylic acids usually leads to new products and commonly reduces dimensionality of the networks formed (Ye *et al.*, 2005). We report here the crystal structure of   a Cd^II^ complex with mixed 2-naphthol-5-carboxylic acid  and 1,10-phenanthroline  as ligands.

The structure of the title complex   consists of a centrosymmetric dinuclear unit and four lattice water molecules. The Cd^II^ center is six-coordinated in an distorted octahedral geometry, by two nitrogen donors atoms from one phenanthroline ligand and four O-atoms from three 2-naphthol-5-carboxylate ligands. For 2-naphthol-5-carboxylate, there exist two different kinds of coordination modes with the Cd^II ^center, namely µ_1_-η^1^:η^1^-chelating and µ_2_-η^2^:η^0^-bridging modes. In this manner two Cd^II^center are connected to form a four-membered ring [Cd(1)–O(3)–Cd(1 A)–O(3 A)] with the Cd(1)···Cd(1 A) separation of 3.671 (1) Å (symmetry operation (A) = 1 - *x*, 1 - *y*, 1 - *z*).

### Experimental

A mixed solution of 2-naphthol-5-carboxylic acid (0.05 mmol) and 1,10-phenanthroline (0.05 mmol) in CH_3_OH (10 ml) in the presence of excess 2,6-dimethylpyridine (*ca *0.05 ml for adjusting the pH value to basic condition) was carefully layered on top of a H_2_O solution (15 ml) of Cd(ClO_4_)_2_ (0.1 mmol) in a test tube. Yellow single crystals suitable for X-ray analysis of the title complex (I) appeared at the tube wall after *ca *three weeks at room temperature. Yield: ~30% based on 2-naphthol-5-carboxylic acid. Elemental analysis calculated for C_68_H_52_Cd_2_N_4_O_16_: C 58.09, H 3.73, N 3.98%; found: C 59.16, H 3.65, N 3.85%. IR (KBr pellet, cm^-1^): 3403 s (br), 2977*m*, 2931*m*, 2770w, 2559w, 1597*m*, 1554*m*, 1521 s, 1468w, 1422*m*, 1377 s, 1302*m*, 1243*m*, 1148*m*, 1100w, 1049w, 953*m*, 847*m*, 791*m*, 767*m*, 725*m*, 663*m*, 604w.

### Refinement

All  H-atoms were refined as riding with   O—H = 0.85Å and C—H = 0.93 Å and *U*_iso_(H) = 1.2 *U*_eq_ (C,O).

### Crystal data

**Table:**

[Cd_2_(C_11_H_7_O_3_)_4_(C_12_H_8_N_2_)_2_]·4H_2_O	*F*(000) = 1424
*M**_r_* = 1405.96	*D*_x_ = 1.611 Mg m^−^^3^
Monoclinic, *P*2_1_/*c*	Mo *K*α radiation, λ = 0.71073 Å
*a* = 11.7382 (11) Å	Cell parameters from 5160 reflections
*b* = 15.1433 (14) Å	θ = 2.4–23.9°
*c* = 18.2059 (13) Å	µ = 0.81 mm^−^^1^
β = 116.430 (4)°	*T* = 296 K
*V* = 2897.9 (4) Å^3^	Block, yellow
*Z* = 2	0.28 × 0.21 × 0.18 mm

### Data collection

**Table:**

Bruker SMART CCD area-detector diffractometer	5096 independent reflections
Radiation source: fine-focus sealed tube	4064 reflections with *I* > 2σ(*I*)
graphite	*R*_int_ = 0.037
φ and ω scans	θ_max_ = 25.0°, θ_min_ = 2.4°
Absorption correction: multi-scan (*SADABS*; Sheldrick, 1996)	*h* = −13→13
*T*_min_ = 0.804, *T*_max_ = 0.868	*k* = −18→18
20423 measured reflections	*l* = −21→21

### Refinement

**Table:**

Refinement on *F*^2^	Primary atom site location: structure-invariant direct methods
Least-squares matrix: full	Secondary atom site location: difference Fourier map
*R*[*F*^2^ > 2σ(*F*^2^)] = 0.028	Hydrogen site location: inferred from neighbouring sites
*wR*(*F*^2^) = 0.063	H-atom parameters constrained
*S* = 1.03	*w* = 1/[σ^2^(*F*_o_^2^) + (0.022*P*)^2^ + 1.6679*P*] where *P* = (*F*_o_^2^ + 2*F*_c_^2^)/3
5096 reflections	(Δ/σ)_max_ = 0.002
406 parameters	Δρ_max_ = 0.30 e Å^−^^3^
0 restraints	Δρ_min_ = −0.40 e Å^−^^3^

### Special details

**Table:**

Geometry. All e.s.d.'s (except the e.s.d. in the dihedral angle between two l.s. planes) are estimated using the full covariance matrix. The cell e.s.d.'s are taken into account individually in the estimation of e.s.d.'s in distances, angles and torsion angles; correlations between e.s.d.'s in cell parameters are only used when they are defined by crystal symmetry. An approximate (isotropic) treatment of cell e.s.d.'s is used for estimating e.s.d.'s involving l.s. planes.
Refinement. Refinement of *F*^2^ against ALL reflections. The weighted *R*-factor *wR* and goodness of fit *S* are based on *F*^2^, conventional *R*-factors *R* are based on *F*, with *F* set to zero for negative *F*^2^. The threshold expression of *F*^2^ > σ(*F*^2^) is used only for calculating *R*-factors(gt) *etc*. and is not relevant to the choice of reflections for refinement. *R*-factors based on *F*^2^ are statistically about twice as large as those based on *F*, and *R*- factors based on ALL data will be even larger.

### Fractional atomic coordinates and isotropic or equivalent isotropic displacement parameters (Å^2^)

**Table:**

	*x*	*y*	*z*	*U*_iso_*/*U*_eq_	
Cd1	0.648118 (18)	0.537570 (13)	0.499232 (12)	0.03367 (7)	
O1	0.64852 (18)	0.68046 (12)	0.45742 (12)	0.0398 (5)	
O2	0.65340 (19)	0.67592 (12)	0.57928 (12)	0.0462 (5)	
O3	0.56800 (17)	0.45469 (13)	0.57326 (11)	0.0410 (5)	
O4	0.51162 (19)	0.46283 (14)	0.67285 (12)	0.0490 (5)	
O5	0.5108 (2)	1.04569 (13)	0.70518 (13)	0.0550 (6)	
H5B	0.5048	1.0182	0.7421	0.082*	
O6	1.0184 (2)	0.17488 (14)	0.63847 (15)	0.0648 (7)	
H6B	1.0940	0.1780	0.6711	0.097*	
N1	0.7308 (2)	0.42210 (15)	0.45474 (14)	0.0359 (5)	
N2	0.8635 (2)	0.52345 (14)	0.59125 (13)	0.0332 (5)	
C1	0.6666 (3)	0.3693 (2)	0.39153 (19)	0.0494 (8)	
H1A	0.5793	0.3778	0.3619	0.059*	
C2	0.7228 (3)	0.3020 (2)	0.3672 (2)	0.0591 (9)	
H2A	0.6736	0.2655	0.3234	0.071*	
C3	0.8509 (3)	0.2903 (2)	0.4086 (2)	0.0570 (9)	
H3A	0.8906	0.2469	0.3920	0.068*	
C4	0.9226 (3)	0.34400 (19)	0.47602 (18)	0.0410 (7)	
C5	1.0570 (3)	0.3349 (2)	0.5234 (2)	0.0498 (8)	
H5A	1.1002	0.2935	0.5075	0.060*	
C6	1.1222 (3)	0.3842 (2)	0.5897 (2)	0.0486 (8)	
H6A	1.2097	0.3767	0.6191	0.058*	
C7	1.0592 (2)	0.44858 (19)	0.61633 (17)	0.0380 (7)	
C8	1.1219 (3)	0.4995 (2)	0.6869 (2)	0.0549 (9)	
H8A	1.2089	0.4925	0.7190	0.066*	
C9	1.0569 (3)	0.5592 (2)	0.7088 (2)	0.0566 (9)	
H9A	1.0981	0.5926	0.7563	0.068*	
C10	0.9269 (3)	0.5697 (2)	0.65892 (18)	0.0433 (7)	
H10A	0.8828	0.6113	0.6740	0.052*	
C11	0.9278 (2)	0.46242 (17)	0.56963 (16)	0.0307 (6)	
C12	0.8583 (3)	0.40913 (17)	0.49782 (16)	0.0323 (6)	
C13	0.6536 (2)	0.71904 (18)	0.52027 (18)	0.0363 (7)	
C14	0.6668 (2)	0.81874 (18)	0.52391 (16)	0.0347 (6)	
C15	0.7268 (3)	0.85596 (19)	0.48148 (17)	0.0386 (7)	
H15A	0.7517	0.8200	0.4498	0.046*	
C16	0.7511 (3)	0.94664 (19)	0.48501 (18)	0.0427 (7)	
H16A	0.7918	0.9704	0.4559	0.051*	
C17	0.7152 (3)	1.0001 (2)	0.53121 (17)	0.0423 (7)	
H17A	0.7340	1.0601	0.5347	0.051*	
C18	0.6500 (2)	0.96592 (18)	0.57379 (16)	0.0340 (6)	
C19	0.6096 (3)	1.02198 (19)	0.61936 (17)	0.0404 (7)	
H19A	0.6262	1.0822	0.6213	0.048*	
C20	0.5467 (3)	0.98935 (19)	0.66048 (17)	0.0398 (7)	
C21	0.5185 (3)	0.89926 (19)	0.65634 (18)	0.0442 (7)	
H21A	0.4743	0.8774	0.6840	0.053*	
C22	0.5550 (3)	0.84284 (19)	0.61223 (18)	0.0410 (7)	
H22A	0.5339	0.7833	0.6095	0.049*	
C23	0.6245 (2)	0.87360 (17)	0.57055 (16)	0.0335 (6)	
C24	0.5938 (3)	0.44834 (18)	0.64928 (17)	0.0367 (7)	
C25	0.7267 (3)	0.42322 (18)	0.70921 (16)	0.0347 (6)	
C26	0.7973 (3)	0.35801 (17)	0.69061 (16)	0.0338 (6)	
C27	0.7474 (3)	0.30337 (18)	0.61980 (18)	0.0424 (7)	
H27A	0.6619	0.3082	0.5827	0.051*	
C28	0.8219 (3)	0.24406 (19)	0.60497 (19)	0.0480 (8)	
H28A	0.7865	0.2093	0.5580	0.058*	
C29	0.9512 (3)	0.23452 (19)	0.6594 (2)	0.0472 (8)	
C30	1.0025 (3)	0.28396 (19)	0.72909 (18)	0.0435 (7)	
H30A	1.0880	0.2771	0.7655	0.052*	
C31	0.9281 (3)	0.34535 (18)	0.74713 (17)	0.0365 (7)	
C32	0.9808 (3)	0.3957 (2)	0.81986 (17)	0.0436 (7)	
H32A	1.0659	0.3879	0.8567	0.052*	
C33	0.9095 (3)	0.4554 (2)	0.83720 (17)	0.0464 (8)	
H33A	0.9455	0.4872	0.8859	0.056*	
C34	0.7818 (3)	0.4687 (2)	0.78147 (17)	0.0426 (7)	
H34A	0.7335	0.5093	0.7939	0.051*	
O1W	0.5418 (2)	0.73556 (15)	0.79947 (13)	0.0609 (6)	
H1	0.5421	0.6825	0.8149	0.073*	
H2	0.5660	0.7580	0.8469	0.073*	
O2W	0.7302 (2)	0.68503 (16)	0.75316 (14)	0.0703 (7)	
H3	0.6802	0.7035	0.7721	0.084*	
H4	0.7056	0.6987	0.7031	0.084*	

### Atomic displacement parameters (Å^2^)

**Table:**

	*U*^11^	*U*^22^	*U*^33^	*U*^12^	*U*^13^	*U*^23^
Cd1	0.02669 (11)	0.03283 (12)	0.04132 (13)	0.00375 (9)	0.01496 (9)	0.00190 (10)
O1	0.0447 (12)	0.0340 (11)	0.0414 (12)	0.0026 (9)	0.0198 (10)	−0.0031 (9)
O2	0.0598 (14)	0.0343 (11)	0.0479 (13)	0.0056 (10)	0.0270 (11)	0.0014 (10)
O3	0.0316 (10)	0.0542 (13)	0.0301 (11)	−0.0029 (9)	0.0075 (9)	0.0117 (9)
O4	0.0383 (12)	0.0630 (14)	0.0435 (12)	0.0081 (11)	0.0163 (10)	−0.0038 (11)
O5	0.0792 (16)	0.0432 (13)	0.0489 (13)	0.0118 (12)	0.0342 (12)	−0.0012 (10)
O6	0.0744 (16)	0.0466 (14)	0.0730 (17)	0.0131 (12)	0.0324 (14)	−0.0038 (12)
N1	0.0318 (13)	0.0312 (13)	0.0397 (14)	0.0015 (10)	0.0115 (11)	0.0006 (11)
N2	0.0292 (12)	0.0337 (13)	0.0361 (13)	0.0014 (10)	0.0139 (10)	−0.0006 (10)
C1	0.0446 (18)	0.0463 (19)	0.0471 (19)	−0.0024 (15)	0.0114 (16)	−0.0044 (16)
C2	0.067 (2)	0.046 (2)	0.056 (2)	−0.0020 (18)	0.0203 (19)	−0.0174 (17)
C3	0.069 (2)	0.0444 (19)	0.061 (2)	0.0089 (18)	0.033 (2)	−0.0127 (17)
C4	0.0452 (18)	0.0364 (16)	0.0466 (18)	0.0077 (14)	0.0252 (15)	0.0014 (14)
C5	0.050 (2)	0.0442 (19)	0.066 (2)	0.0179 (16)	0.0366 (18)	0.0073 (17)
C6	0.0339 (17)	0.057 (2)	0.058 (2)	0.0144 (15)	0.0232 (16)	0.0130 (17)
C7	0.0286 (15)	0.0434 (17)	0.0413 (17)	0.0048 (13)	0.0150 (13)	0.0091 (14)
C8	0.0307 (16)	0.073 (2)	0.048 (2)	0.0028 (16)	0.0065 (15)	−0.0003 (18)
C9	0.0446 (19)	0.070 (2)	0.0421 (19)	−0.0030 (17)	0.0071 (16)	−0.0167 (17)
C10	0.0415 (18)	0.0455 (17)	0.0406 (18)	0.0021 (14)	0.0163 (15)	−0.0077 (14)
C11	0.0273 (14)	0.0303 (14)	0.0359 (15)	0.0041 (12)	0.0153 (12)	0.0036 (13)
C12	0.0336 (15)	0.0288 (14)	0.0366 (15)	0.0026 (12)	0.0175 (13)	0.0048 (13)
C13	0.0286 (15)	0.0334 (16)	0.0427 (18)	0.0056 (12)	0.0121 (14)	0.0006 (14)
C14	0.0280 (14)	0.0348 (15)	0.0349 (16)	0.0049 (12)	0.0083 (13)	0.0021 (12)
C15	0.0352 (16)	0.0419 (17)	0.0360 (16)	0.0018 (13)	0.0134 (13)	0.0005 (13)
C16	0.0426 (17)	0.0462 (19)	0.0410 (17)	−0.0068 (14)	0.0202 (15)	0.0031 (14)
C17	0.0462 (18)	0.0326 (15)	0.0426 (18)	−0.0050 (14)	0.0148 (15)	0.0028 (14)
C18	0.0309 (14)	0.0323 (15)	0.0297 (14)	0.0017 (13)	0.0053 (12)	0.0023 (13)
C19	0.0437 (17)	0.0351 (17)	0.0333 (16)	0.0014 (14)	0.0091 (14)	0.0011 (13)
C20	0.0432 (17)	0.0392 (17)	0.0327 (16)	0.0085 (14)	0.0130 (14)	0.0006 (13)
C21	0.0475 (18)	0.0427 (18)	0.0476 (18)	0.0024 (15)	0.0259 (15)	0.0053 (15)
C22	0.0408 (17)	0.0339 (16)	0.0490 (18)	0.0008 (13)	0.0206 (15)	0.0017 (14)
C23	0.0309 (15)	0.0321 (15)	0.0309 (15)	0.0026 (12)	0.0078 (12)	0.0038 (12)
C24	0.0340 (16)	0.0339 (16)	0.0351 (16)	−0.0029 (13)	0.0091 (13)	0.0011 (13)
C25	0.0339 (15)	0.0378 (15)	0.0297 (15)	0.0010 (13)	0.0118 (13)	0.0065 (13)
C26	0.0359 (15)	0.0317 (15)	0.0300 (15)	−0.0030 (12)	0.0111 (13)	0.0048 (12)
C27	0.0440 (17)	0.0339 (16)	0.0391 (17)	−0.0035 (14)	0.0095 (14)	0.0031 (14)
C28	0.062 (2)	0.0311 (16)	0.0436 (18)	−0.0045 (15)	0.0170 (17)	−0.0027 (14)
C29	0.062 (2)	0.0304 (16)	0.055 (2)	0.0074 (15)	0.0315 (18)	0.0066 (15)
C30	0.0400 (17)	0.0386 (17)	0.0448 (19)	0.0066 (14)	0.0124 (15)	0.0089 (15)
C31	0.0370 (16)	0.0338 (16)	0.0342 (16)	0.0014 (13)	0.0119 (13)	0.0077 (13)
C32	0.0370 (16)	0.0505 (19)	0.0340 (16)	0.0031 (15)	0.0076 (14)	0.0050 (14)
C33	0.0462 (18)	0.055 (2)	0.0288 (15)	−0.0015 (16)	0.0087 (14)	−0.0054 (15)
C34	0.0422 (17)	0.0482 (18)	0.0359 (16)	0.0041 (15)	0.0161 (14)	0.0001 (15)
O1W	0.0763 (16)	0.0583 (15)	0.0430 (13)	−0.0083 (13)	0.0220 (12)	−0.0003 (11)
O2W	0.0676 (16)	0.0829 (18)	0.0647 (16)	0.0084 (14)	0.0332 (13)	0.0077 (14)

### Geometric parameters (Å, °)

**Table:**

Cd1—O3^i^	2.2827 (18)	C11—C12	1.441 (4)
Cd1—O1	2.2945 (18)	C13—C14	1.516 (4)
Cd1—N1	2.314 (2)	C14—C15	1.377 (4)
Cd1—O3	2.3247 (18)	C14—C23	1.426 (4)
Cd1—N2	2.339 (2)	C15—C16	1.398 (4)
Cd1—O2	2.5370 (19)	C15—H15A	0.9300
Cd1—C13	2.771 (3)	C16—C17	1.363 (4)
Cd1—O4^i^	2.846 (2)	C16—H16A	0.9300
Cd1—Cd1^i^	3.6706 (5)	C17—C18	1.408 (4)
O1—C13	1.262 (3)	C17—H17A	0.9300
O2—C13	1.258 (3)	C18—C19	1.409 (4)
O3—C24	1.282 (3)	C18—C23	1.425 (4)
O3—Cd1^i^	2.2827 (18)	C19—C20	1.357 (4)
O4—C24	1.238 (3)	C19—H19A	0.9300
O4—Cd1^i^	2.846 (2)	C20—C21	1.398 (4)
O5—C20	1.369 (3)	C21—C22	1.366 (4)
O5—H5B	0.8200	C21—H21A	0.9300
O6—C29	1.360 (3)	C22—C23	1.418 (4)
O6—H6B	0.8200	C22—H22A	0.9300
N1—C1	1.326 (4)	C24—C25	1.501 (4)
N1—C12	1.360 (3)	C25—C34	1.366 (4)
N2—C10	1.322 (3)	C25—C26	1.424 (4)
N2—C11	1.358 (3)	C26—C27	1.420 (4)
C1—C2	1.389 (4)	C26—C31	1.431 (4)
C1—H1A	0.9300	C27—C28	1.362 (4)
C2—C3	1.361 (4)	C27—H27A	0.9300
C2—H2A	0.9300	C28—C29	1.403 (4)
C3—C4	1.399 (4)	C28—H28A	0.9300
C3—H3A	0.9300	C29—C30	1.362 (4)
C4—C12	1.402 (4)	C30—C31	1.411 (4)
C4—C5	1.428 (4)	C30—H30A	0.9300
C5—C6	1.334 (4)	C31—C32	1.410 (4)
C5—H5A	0.9300	C32—C33	1.360 (4)
C6—C7	1.432 (4)	C32—H32A	0.9300
C6—H6A	0.9300	C33—C34	1.402 (4)
C7—C8	1.394 (4)	C33—H33A	0.9300
C7—C11	1.406 (4)	C34—H34A	0.9300
C8—C9	1.353 (4)	O1W—H1	0.8500
C8—H8A	0.9300	O1W—H2	0.8500
C9—C10	1.395 (4)	O2W—H3	0.8499
C9—H9A	0.9300	O2W—H4	0.8502
C10—H10A	0.9300		
			
O3^i^—Cd1—O1	85.73 (7)	C9—C10—H10A	118.5
O3^i^—Cd1—N1	111.57 (8)	N2—C11—C7	121.9 (2)
O1—Cd1—N1	122.24 (7)	N2—C11—C12	118.7 (2)
O3^i^—Cd1—O3	74.38 (7)	C7—C11—C12	119.4 (2)
O1—Cd1—O3	139.69 (7)	N1—C12—C4	122.1 (3)
N1—Cd1—O3	97.80 (8)	N1—C12—C11	118.4 (2)
O3^i^—Cd1—N2	170.86 (7)	C4—C12—C11	119.5 (2)
O1—Cd1—N2	99.36 (7)	O2—C13—O1	121.1 (3)
N1—Cd1—N2	72.17 (8)	O2—C13—C14	121.2 (3)
O3—Cd1—N2	97.04 (7)	O1—C13—C14	117.6 (3)
O3^i^—Cd1—O2	91.52 (7)	O2—C13—Cd1	66.08 (15)
O1—Cd1—O2	53.75 (7)	O1—C13—Cd1	55.04 (14)
N1—Cd1—O2	156.67 (7)	C14—C13—Cd1	171.5 (2)
O3—Cd1—O2	91.47 (7)	C15—C14—C23	119.8 (3)
N2—Cd1—O2	85.51 (7)	C15—C14—C13	116.8 (3)
O3^i^—Cd1—C13	88.87 (7)	C23—C14—C13	123.4 (3)
O1—Cd1—C13	26.80 (7)	C14—C15—C16	121.4 (3)
N1—Cd1—C13	144.11 (8)	C14—C15—H15A	119.3
O3—Cd1—C13	116.46 (8)	C16—C15—H15A	119.3
N2—Cd1—C13	92.32 (8)	C17—C16—C15	119.9 (3)
O2—Cd1—C13	26.95 (7)	C17—C16—H16A	120.0
O3^i^—Cd1—O4^i^	49.13 (6)	C15—C16—H16A	120.0
O1—Cd1—O4^i^	74.60 (6)	C16—C17—C18	121.0 (3)
N1—Cd1—O4^i^	77.68 (7)	C16—C17—H17A	119.5
O3—Cd1—O4^i^	113.50 (6)	C18—C17—H17A	119.5
N2—Cd1—O4^i^	139.51 (7)	C17—C18—C19	120.7 (3)
O2—Cd1—O4^i^	118.02 (6)	C17—C18—C23	119.6 (3)
C13—Cd1—O4^i^	96.75 (7)	C19—C18—C23	119.7 (3)
O3^i^—Cd1—Cd1^i^	37.58 (5)	C20—C19—C18	120.9 (3)
O1—Cd1—Cd1^i^	115.89 (5)	C20—C19—H19A	119.5
N1—Cd1—Cd1^i^	108.34 (6)	C18—C19—H19A	119.5
O3—Cd1—Cd1^i^	36.79 (4)	C19—C20—O5	119.2 (3)
N2—Cd1—Cd1^i^	133.75 (5)	C19—C20—C21	120.0 (3)
O2—Cd1—Cd1^i^	91.87 (5)	O5—C20—C21	120.8 (3)
C13—Cd1—Cd1^i^	105.66 (6)	C22—C21—C20	120.9 (3)
O4^i^—Cd1—Cd1^i^	81.11 (4)	C22—C21—H21A	119.5
C13—O1—Cd1	98.15 (17)	C20—C21—H21A	119.5
C13—O2—Cd1	86.96 (16)	C21—C22—C23	121.0 (3)
C24—O3—Cd1^i^	107.21 (16)	C21—C22—H22A	119.5
C24—O3—Cd1	134.76 (17)	C23—C22—H22A	119.5
Cd1^i^—O3—Cd1	105.62 (7)	C22—C23—C18	117.4 (3)
C24—O4—Cd1^i^	81.45 (16)	C22—C23—C14	124.3 (3)
C20—O5—H5B	109.5	C18—C23—C14	118.3 (3)
C29—O6—H6B	109.5	O4—C24—O3	121.0 (3)
C1—N1—C12	117.9 (2)	O4—C24—C25	120.7 (3)
C1—N1—Cd1	126.5 (2)	O3—C24—C25	118.3 (2)
C12—N1—Cd1	115.64 (17)	C34—C25—C26	120.2 (3)
C10—N2—C11	118.4 (2)	C34—C25—C24	117.8 (3)
C10—N2—Cd1	126.87 (19)	C26—C25—C24	122.0 (2)
C11—N2—Cd1	114.69 (16)	C27—C26—C25	124.9 (3)
N1—C1—C2	123.5 (3)	C27—C26—C31	116.8 (3)
N1—C1—H1A	118.3	C25—C26—C31	118.3 (2)
C2—C1—H1A	118.3	C28—C27—C26	121.5 (3)
C3—C2—C1	119.0 (3)	C28—C27—H27A	119.2
C3—C2—H2A	120.5	C26—C27—H27A	119.2
C1—C2—H2A	120.5	C27—C28—C29	121.1 (3)
C2—C3—C4	119.6 (3)	C27—C28—H28A	119.4
C2—C3—H3A	120.2	C29—C28—H28A	119.4
C4—C3—H3A	120.2	O6—C29—C30	123.7 (3)
C3—C4—C12	117.9 (3)	O6—C29—C28	116.8 (3)
C3—C4—C5	123.0 (3)	C30—C29—C28	119.5 (3)
C12—C4—C5	119.1 (3)	C29—C30—C31	121.1 (3)
C6—C5—C4	121.9 (3)	C29—C30—H30A	119.4
C6—C5—H5A	119.1	C31—C30—H30A	119.4
C4—C5—H5A	119.1	C32—C31—C30	121.1 (3)
C5—C6—C7	120.8 (3)	C32—C31—C26	119.0 (3)
C5—C6—H6A	119.6	C30—C31—C26	119.9 (3)
C7—C6—H6A	119.6	C33—C32—C31	121.3 (3)
C8—C7—C11	117.5 (3)	C33—C32—H32A	119.4
C8—C7—C6	123.2 (3)	C31—C32—H32A	119.4
C11—C7—C6	119.3 (3)	C32—C33—C34	119.9 (3)
C9—C8—C7	120.4 (3)	C32—C33—H33A	120.1
C9—C8—H8A	119.8	C34—C33—H33A	120.1
C7—C8—H8A	119.8	C25—C34—C33	121.3 (3)
C8—C9—C10	118.8 (3)	C25—C34—H34A	119.3
C8—C9—H9A	120.6	C33—C34—H34A	119.3
C10—C9—H9A	120.6	H1—O1W—H2	95.3
N2—C10—C9	123.1 (3)	H3—O2W—H4	112.8
N2—C10—H10A	118.5		
			
O3^i^—Cd1—O1—C13	95.96 (16)	C3—C4—C12—N1	−1.3 (4)
N1—Cd1—O1—C13	−151.16 (15)	C5—C4—C12—N1	179.1 (3)
O3—Cd1—O1—C13	36.3 (2)	C3—C4—C12—C11	176.6 (3)
N2—Cd1—O1—C13	−76.40 (17)	C5—C4—C12—C11	−3.0 (4)
O2—Cd1—O1—C13	0.94 (15)	N2—C11—C12—N1	−0.2 (4)
O4^i^—Cd1—O1—C13	144.66 (17)	C7—C11—C12—N1	178.4 (2)
Cd1^i^—Cd1—O1—C13	72.82 (16)	N2—C11—C12—C4	−178.1 (2)
O3^i^—Cd1—O2—C13	−84.52 (16)	C7—C11—C12—C4	0.4 (4)
O1—Cd1—O2—C13	−0.94 (15)	Cd1—O2—C13—O1	1.6 (3)
N1—Cd1—O2—C13	87.3 (2)	Cd1—O2—C13—C14	−175.2 (2)
O3—Cd1—O2—C13	−158.93 (16)	Cd1—O1—C13—O2	−1.8 (3)
N2—Cd1—O2—C13	104.13 (16)	Cd1—O1—C13—C14	175.09 (19)
O4^i^—Cd1—O2—C13	−41.20 (17)	O3^i^—Cd1—C13—O2	95.57 (16)
Cd1^i^—Cd1—O2—C13	−122.12 (15)	O1—Cd1—C13—O2	178.3 (3)
O3^i^—Cd1—O3—C24	−135.2 (3)	N1—Cd1—C13—O2	−137.55 (16)
O1—Cd1—O3—C24	−71.9 (3)	O3—Cd1—C13—O2	23.67 (17)
N1—Cd1—O3—C24	114.5 (3)	N2—Cd1—C13—O2	−75.37 (16)
N2—Cd1—O3—C24	41.6 (3)	O4^i^—Cd1—C13—O2	144.16 (15)
O2—Cd1—O3—C24	−44.1 (3)	Cd1^i^—Cd1—C13—O2	61.53 (16)
C13—Cd1—O3—C24	−54.6 (3)	O3^i^—Cd1—C13—O1	−82.76 (16)
O4^i^—Cd1—O3—C24	−165.6 (2)	N1—Cd1—C13—O1	44.1 (2)
Cd1^i^—Cd1—O3—C24	−135.2 (3)	O3—Cd1—C13—O1	−154.66 (15)
O3^i^—Cd1—O3—Cd1^i^	0.0	N2—Cd1—C13—O1	106.30 (16)
O1—Cd1—O3—Cd1^i^	63.32 (13)	O2—Cd1—C13—O1	−178.3 (3)
N1—Cd1—O3—Cd1^i^	−110.30 (9)	O4^i^—Cd1—C13—O1	−34.17 (16)
N2—Cd1—O3—Cd1^i^	176.82 (8)	Cd1^i^—Cd1—C13—O1	−116.79 (15)
O2—Cd1—O3—Cd1^i^	91.16 (8)	O2—C13—C14—C15	148.9 (3)
C13—Cd1—O3—Cd1^i^	80.68 (9)	O1—C13—C14—C15	−28.0 (4)
O4^i^—Cd1—O3—Cd1^i^	−30.40 (10)	O2—C13—C14—C23	−28.9 (4)
O3^i^—Cd1—N1—C1	4.9 (3)	O1—C13—C14—C23	154.2 (3)
O1—Cd1—N1—C1	−94.0 (2)	C23—C14—C15—C16	2.0 (4)
O3—Cd1—N1—C1	81.1 (2)	C13—C14—C15—C16	−175.9 (3)
N2—Cd1—N1—C1	176.0 (3)	C14—C15—C16—C17	0.0 (4)
O2—Cd1—N1—C1	−166.4 (2)	C15—C16—C17—C18	−2.0 (4)
C13—Cd1—N1—C1	−115.8 (2)	C16—C17—C18—C19	−178.0 (3)
O4^i^—Cd1—N1—C1	−31.4 (2)	C16—C17—C18—C23	1.9 (4)
Cd1^i^—Cd1—N1—C1	44.8 (2)	C17—C18—C19—C20	180.0 (3)
O3^i^—Cd1—N1—C12	−176.18 (17)	C23—C18—C19—C20	0.1 (4)
O1—Cd1—N1—C12	84.91 (19)	C18—C19—C20—O5	178.9 (2)
O3—Cd1—N1—C12	−99.96 (18)	C18—C19—C20—C21	−1.6 (4)
N2—Cd1—N1—C12	−5.04 (18)	C19—C20—C21—C22	0.9 (4)
O2—Cd1—N1—C12	12.6 (3)	O5—C20—C21—C22	−179.6 (3)
C13—Cd1—N1—C12	63.1 (2)	C20—C21—C22—C23	1.2 (4)
O4^i^—Cd1—N1—C12	147.59 (19)	C21—C22—C23—C18	−2.5 (4)
Cd1^i^—Cd1—N1—C12	−136.24 (17)	C21—C22—C23—C14	179.5 (3)
O1—Cd1—N2—C10	61.9 (2)	C17—C18—C23—C22	−178.0 (3)
N1—Cd1—N2—C10	−177.1 (2)	C19—C18—C23—C22	1.9 (4)
O2—Cd1—N2—C10	9.8 (2)	C17—C18—C23—C14	0.2 (4)
C13—Cd1—N2—C10	35.9 (2)	C19—C18—C23—C14	−180.0 (2)
O4^i^—Cd1—N2—C10	139.1 (2)	C15—C14—C23—C22	176.0 (3)
Cd1^i^—Cd1—N2—C10	−78.5 (2)	C13—C14—C23—C22	−6.3 (4)
O1—Cd1—N2—C11	−116.08 (18)	C15—C14—C23—C18	−2.1 (4)
N1—Cd1—N2—C11	4.91 (17)	C13—C14—C23—C18	175.7 (2)
O2—Cd1—N2—C11	−168.20 (18)	Cd1^i^—O4—C24—O3	9.9 (2)
C13—Cd1—N2—C11	−142.10 (18)	Cd1^i^—O4—C24—C25	−170.3 (3)
O4^i^—Cd1—N2—C11	−38.9 (2)	Cd1^i^—O3—C24—O4	−12.9 (3)
Cd1^i^—Cd1—N2—C11	103.51 (17)	Cd1—O3—C24—O4	121.9 (3)
C12—N1—C1—C2	−0.1 (4)	Cd1^i^—O3—C24—C25	167.38 (19)
Cd1—N1—C1—C2	178.8 (2)	Cd1—O3—C24—C25	−57.8 (4)
N1—C1—C2—C3	−1.8 (5)	O4—C24—C25—C34	−43.3 (4)
C1—C2—C3—C4	2.1 (5)	O3—C24—C25—C34	136.4 (3)
C2—C3—C4—C12	−0.7 (5)	O4—C24—C25—C26	140.3 (3)
C2—C3—C4—C5	178.9 (3)	O3—C24—C25—C26	−40.0 (4)
C3—C4—C5—C6	−176.8 (3)	C34—C25—C26—C27	176.1 (3)
C12—C4—C5—C6	2.8 (5)	C24—C25—C26—C27	−7.6 (4)
C4—C5—C6—C7	0.2 (5)	C34—C25—C26—C31	−3.4 (4)
C5—C6—C7—C8	177.5 (3)	C24—C25—C26—C31	172.8 (2)
C5—C6—C7—C11	−2.8 (4)	C25—C26—C27—C28	178.1 (3)
C11—C7—C8—C9	0.5 (5)	C31—C26—C27—C28	−2.4 (4)
C6—C7—C8—C9	−179.9 (3)	C26—C27—C28—C29	0.1 (4)
C7—C8—C9—C10	−1.1 (5)	C27—C28—C29—O6	−178.7 (3)
C11—N2—C10—C9	0.4 (4)	C27—C28—C29—C30	1.5 (5)
Cd1—N2—C10—C9	−177.5 (2)	O6—C29—C30—C31	179.5 (3)
C8—C9—C10—N2	0.7 (5)	C28—C29—C30—C31	−0.7 (4)
C10—N2—C11—C7	−1.0 (4)	C29—C30—C31—C32	179.1 (3)
Cd1—N2—C11—C7	177.1 (2)	C29—C30—C31—C26	−1.6 (4)
C10—N2—C11—C12	177.4 (2)	C27—C26—C31—C32	−177.7 (2)
Cd1—N2—C11—C12	−4.4 (3)	C25—C26—C31—C32	1.9 (4)
C8—C7—C11—N2	0.6 (4)	C27—C26—C31—C30	3.1 (4)
C6—C7—C11—N2	−179.0 (3)	C25—C26—C31—C30	−177.3 (2)
C8—C7—C11—C12	−177.9 (3)	C30—C31—C32—C33	179.6 (3)
C6—C7—C11—C12	2.5 (4)	C26—C31—C32—C33	0.3 (4)
C1—N1—C12—C4	1.7 (4)	C31—C32—C33—C34	−1.1 (5)
Cd1—N1—C12—C4	−177.4 (2)	C26—C25—C34—C33	2.7 (4)
C1—N1—C12—C11	−176.2 (2)	C24—C25—C34—C33	−173.7 (3)
Cd1—N1—C12—C11	4.7 (3)	C32—C33—C34—C25	−0.4 (5)

Symmetry codes: (i) −*x*+1, −*y*+1, −*z*+1.

### Hydrogen-bond geometry (Å, °)

**Table:**

*D*—H···*A*	*D*—H	H···*A*	*D*···*A*	*D*—H···*A*
O1W—H1···O5^ii^	0.85	2.15	2.934 (3)	154
O1W—H2···O1^iii^	0.85	2.03	2.872 (3)	170
O2W—H3···O1W	0.85	1.96	2.801 (4)	170
O2W—H4···O2	0.85	2.08	2.883 (3)	156
O5—H5B···O4^iv^	0.82	1.84	2.664 (3)	176
O6—H6B···O2W^v^	0.82	1.91	2.728 (4)	180

Symmetry codes: (ii) −*x*+1, *y*−1/2, −*z*+3/2; (iii) *x*, −*y*+3/2, *z*+1/2; (iv) −*x*+1, *y*+1/2, −*z*+3/2; (v) −*x*+2, *y*−1/2, −*z*+3/2.
